# Maternal Iodine Status During Pregnancy and Child Neurodevelopment: A Systematic Review and Dose–Response Meta-Analysis of Prospective Cohort Studies

**DOI:** 10.3390/nu18091474

**Published:** 2026-05-05

**Authors:** Qingshan Luo, Zhou Wang, Jie Jiang, Xianru Luo, Tingxuan Feng, Chao Wang, Shasha Ruan, Xiaoheng Li

**Affiliations:** 1 Division of Nutrition and Food Safety, Shenzhen Center for Disease Control and Prevention, Shenzhen 518000, China; lqssky1@126.com (Q.L.); wangzhou861@126.com (Z.W.); szjiangjie@139.com (J.J.); luoxianru16@163.com (X.L.); fengtingxuan1996@163.com (T.F.); raulw2003@163.com (C.W.); ruanshasha1105@163.com (S.R.); 2 Shenzhen Center for Disease Control and Prevention, Shenzhen 518000, China

**Keywords:** iodine, pregnancy, neurodevelopment, dose–response, meta-analysis, cohort study

## Abstract

Background: Iodine deficiency during pregnancy remains a leading cause of preventable neurodevelopmental impairment worldwide, yet quantitative characterization of the dose–response relationship between maternal iodine status and child neurodevelopment is lacking. Methods: A systematic search of PubMed, Embase, the Cochrane Library, and Web of Science was conducted for prospective cohort studies published up to February 2026 reporting associations between maternal iodine status and child neurodevelopmental outcomes across at least three exposure categories. All continuous effect estimates were converted to standardized mean differences (Hedges’ g) to permit pooling across heterogeneous assessment instruments, and meta-analyses were stratified by neurodevelopmental domain (cognitive, language, motor, behavior, academic). A two-stage dose–response meta-analysis was used to characterize non-linearity. Risk of bias was evaluated using the Newcastle–Ottawa Scale (NOS). Results: Ten prospective cohort publications corresponding to eight independent cohorts were included. After converting all continuous effect estimates to standardized mean differences (Hedges’ g) and consolidating the three overlapping MoBa publications into a single cohort, the pooled analysis revealed a significant negative association between suboptimal maternal iodine status and child neurodevelopmental performance (Hedges’ g = −0.13, 95% CI: −0.20 to −0.06, *p* < 0.001; *I*^2^ = 95.2%). Domain-stratified analysis identified cognitive outcomes as most consistently affected (g = −0.22, 95% CI: −0.30 to −0.14; *I*^2^ = 37.5%), followed by motor (g = −0.17; *I*^2^ = 0%) and language outcomes (g = −0.16; *I*^2^ = 92.5%), with between-domain heterogeneity explaining 38.6% of the total variance (*p* = 0.012). Binary outcome analysis confirmed increased odds of adverse neurodevelopmental outcomes (OR = 1.19, 95% CI: 1.03 to 1.39, *p* = 0.026). Subgroup analysis by iodine exposure indicator showed directionally consistent negative effects across dietary intake (g = −0.11), UIC (g = −0.11) and UI/Cr (g = −0.28), with no significant between-subgroup difference (*p* = 0.237). Exploratory dose–response modeling on the Hedges’ g scale suggested that neurodevelopmental performance in the fitted curves approached its maximum within a mid-range of dietary iodine intake (approximately 150–300 µg/d); however, the quadratic non-linearity terms did not reach statistical significance after cohort consolidation (*p* = 0.612 for dietary intake; *p* = 0.436 for UI/Cr), and these findings should therefore be interpreted as exploratory. Conclusions: Suboptimal maternal iodine status during pregnancy was associated with modest decrements in child neurodevelopmental performance, with exploratory dose–response analyses suggesting that the fitted curves approached their maximum within a mid-range of dietary iodine intake. Although statistical evidence for quadratic non-linearity was attenuated after consolidating overlapping cohorts, the directional pattern across indicators remained consistent with an inverted U-shaped relationship, supporting maintenance of adequate but not excessive iodine nutrition during pregnancy.

## 1. Introduction

Iodine is an essential trace element required for the synthesis of thyroid hormones, thyroxine (T4) and triiodothyronine (T3), which play an indispensable role in fetal brain development [1,2]. During pregnancy, iodine requirements increase by approximately 50% to support enhanced maternal thyroid hormone production, greater renal iodine clearance, and placental transfer of iodine to the fetus [3]. Inadequate iodine nutrition during this critical window can result in maternal hypothyroxinemia, which in turn compromises the supply of T4 to the developing fetal brain, a process that is particularly consequential before the onset of fetal thyroid function at approximately 18–20 weeks of gestation [4,5]. The resultant deficits in neurodevelopment range from severe forms such as cretinism to subtler impairments in cognitive function, language acquisition, and academic performance that may persist into adulthood [6,7].

In response to these concerns, the WHO, the American Thyroid Association (ATA), and the European Thyroid Association (ETA) have issued recommendations for iodine supplementation during pregnancy, generally advising a daily intake of 250 μg/d [8,9,10]. However, these guidelines exhibit considerable discordance regarding the threshold for recommending supplementation and the populations to whom such recommendations should apply. The WHO recommends supplementation only in regions where household access to iodized salt falls below 90%, while the ATA recommends 150 μg/d of supplemental iodine for all pregnant women in North America regardless of background iodine status [10,11]. Moreover, the optimal upper limit of iodine intake during pregnancy remains contentious, as emerging evidence suggests that excessive iodine may also impair thyroid function and fetal neurodevelopment through the Wolff–Chaikoff effect [12,13]. This lack of harmonized, evidence-based guidance underscores the urgent need for quantitative data characterizing the precise relationship between maternal iodine levels and child neurodevelopmental outcomes.

Critically, prior meta-analyses in this field have been constrained by several methodological limitations. Most have pooled studies using simple binary or categorical comparisons (e.g., deficient versus sufficient) without modeling the continuous dose–response relationship, thereby potentially obscuring important nonlinear patterns such as threshold effects or adverse consequences of excessive intake [14,15,16]. Furthermore, previous reviews have frequently combined randomized controlled trials (RCTs) with observational studies without adequately addressing fundamental differences in study design, or have failed to account for heterogeneity arising from different iodine exposure indicators (e.g., UIC versus dietary intake from food frequency questionnaires) [17]. None, to our knowledge, have employed restricted cubic spline modeling within a two-stage dose–response meta-analytic framework to characterize the shape of the association between maternal iodine level and child neurodevelopment using data exclusively from prospective cohort studies.

To address these gaps, the present systematic review and dose–response meta-analysis aims to (1) quantify the overall association between maternal iodine status during pregnancy and child neurodevelopmental outcomes based on prospective cohort studies; (2) explore sources of between-study heterogeneity through subgroup analyses stratified by iodine exposure indicator; (3) characterize the dose–response relationship using linear, quadratic, and restricted cubic spline models to identify potential nonlinear patterns including threshold effects and optimal iodine ranges; and (4) provide evidence-based insights to inform clinical guidelines and public health policy regarding iodine nutrition during pregnancy.

## 2. Methods

This systematic review and dose–response meta-analysis was conducted in accordance with the Preferred Reporting Items for Systematic Reviews and Meta-Analyses (PRISMA) 2020 guidelines. The study protocol was prospectively registered in the International Prospective Register of Systematic Reviews (PROSPERO; registration number: CRD420261341886). No amendments were made to the registered protocol.

### 2.1. Inclusion and Exclusion Criteria

Studies were selected according to a predefined set of eligibility criteria based on the Population, Exposure, Comparator, Outcome, and Study design (PECOS) framework, as described below.

*Population*: Eligible studies enrolled pregnant women who underwent assessment of iodine nutritional status during gestation and their live-born singleton offspring.

*Exposure*: The exposure of interest was maternal iodine nutritional status during pregnancy. Two categories of measurement were accepted: (i) urinary iodine biomarkers, including spot or 24 h urinary iodine concentration (UIC, μg/L) and iodine-to-creatinine ratio (I:Cr, μg/g creatinine), and (ii) estimated dietary iodine intake (μg/d) derived from validated food frequency questionnaires (FFQs).

*Comparator*: Because we adopted a dose–response analytical framework in this review, no single reference group was designated across studies. Instead, each eligible study was required to report results for at least three quantitative categories of maternal iodine exposure (e.g., tertiles, quartiles, or predefined cut-offs), thereby enabling the estimation of a continuous dose–response function.

*Outcomes*: The primary outcomes were measures of child neurodevelopment, encompassing cognitive or intellectual ability, language development, motor development (gross and fine), academic performance, and behavioral outcomes.

*Study design*: Only prospective cohort studies were eligible for inclusion.

Additional exclusion criteria included (i) studies that did not report iodine exposure in at least three quantitative categories; (ii) studies that assessed iodine status solely through thyroid hormone levels without concurrent measurement of urinary iodine or dietary iodine intake; (iii) studies with multiple gestations or that did not distinguish singleton from multiple births in their analyses; and (iv) studies that assessed only neonatal outcomes (e.g., Apgar scores, birth anthropometry) without postnatal neurodevelopmental follow-up.

### 2.2. Search Strategy

A comprehensive literature search was conducted across four electronic databases from inception through 1 February 2026: PubMed, Embase, the Cochrane Library, and Web of Science. Embase and the Cochrane Library were searched simultaneously via the Ovid platform. The search strategy combined Medical Subject Headings (MeSH) and Embase subject headings (Emtree) with free-text terms related to two core concepts: (i) pregnancy and maternal iodine exposure (e.g., “iodine”, “iodide”, “urinary iodine”, “iodine intake”, “iodine status”); and (ii) child neurodevelopment (e.g., “neurodevelopment”, “cognition”, “intelligence”, “language development”, “motor skills”, “behaviour”). Boolean operators (AND, OR) were used to combine concept groups. No date or language restrictions were applied at the search stage. The complete search strategies for each database are provided in Supplementary File S1.

In addition to the electronic database search, the reference lists of all included studies and relevant review articles were manually screened to identify further eligible publications. The cited and citing references of key studies were also examined through Google Scholar.

### 2.3. Study Selection and Data Extraction

Following the removal of duplicate records, titles and abstracts were screened independently by two reviewers against the predefined eligibility criteria. Full-text articles of potentially relevant studies were then retrieved and assessed for inclusion by the same two reviewers working independently. Disagreements at each stage were resolved through discussion or adjudication by a third reviewer. No automation tools were used in the selection process. Data were extracted independently by two reviewers using a standardized, pilot-tested form. Per study, the following was recorded: first author, year, country, cohort, sample size, maternal age, population iodine background, iodized salt policy, iodine exposure indicator, exposure category definitions, reference category, child age at assessment, neurodevelopmental outcome domains and tools, effect estimates with 95% confidence intervals or standard errors, and covariates adjusted in multivariable models. The midpoint of each category served as the assigned dose; for open-ended categories, the dose was estimated as 1.5 times the lower boundary. Disagreements were resolved by a third reviewer.

Where multiple publications reported overlapping outcome data from the same cohort, only the publication reporting the most comprehensive or most recent follow-up was retained; publications reporting non-overlapping neurodevelopmental domains from the same cohort were retained and subsequently consolidated at the analysis stage.

### 2.4. Risk-of-Bias Assessment

The methodological quality of each included cohort study was assessed independently by two reviewers using the Newcastle–Ottawa Scale (NOS) for cohort studies. The NOS evaluates three domains: selection of study groups (representativeness of the exposed cohort, selection of the non-exposed cohort, ascertainment of exposure, and demonstration that the outcome of interest was not present at the start of the study; maximum 4 stars), comparability of cohorts (control for the most important confounders; maximum 2 stars), and outcome (assessment of outcome, adequacy of follow-up duration, and adequacy of follow-up rate; maximum 3 stars). Total scores range from 0 to 9, with higher scores indicating better methodological quality. Studies scoring 7–9 were considered high-quality (low risk of bias), those scoring 4–6 as moderate-quality, and those scoring 0–3 as low-quality (high risk of bias). Any disagreements were resolved through discussion with a third reviewer.

### 2.5. Statistical Analysis

All analyses were performed using R version 4.5.2; two-sided *p* < 0.05 was considered significant. To permit valid pooling across heterogeneous neurodevelopmental assessment instruments (Bayley-III, WPPSI-III, WISC-IV, MSCA, z-score-transformed outcomes), all continuous effect estimates were converted to standardized mean differences (Hedges’ g). Regression coefficients on z-score-transformed outcomes were interpreted directly as standardized effects; those on raw-score scales were divided by the instrument-specific population standard deviation, with Hedges’ small-sample correction applied throughout. For scales on which higher scores denote greater impairment (ADHD symptoms, reading/writing difficulty z-scores), the sign convention was preserved and interpreted against a positive direction being unfavorable. Detailed formulas are provided in Supplementary File S2.

One primary outcome per cohort–domain combination was pre-specified for the main analysis; all estimates were retained with cluster-robust variance estimation at the cohort level as sensitivity analysis. Random-effects meta-analyses using REML were stratified by neurodevelopmental domain (cognitive, language, motor, behavior, academic); between-domain heterogeneity was assessed via meta-regression. Subgroup analyses by exposure indicator (dietary intake, UIC, UI/Cr), leave-one-out sensitivity analyses, Egger’s test, and trim-and-fill were conducted.

A two-stage dose–response meta-analysis was performed on the Hedges’ g scale. In the first stage, study-specific trends were estimated using generalized least squares regression, with within-study correlation between exposure categories reconstructed via the Greenland–Longnecker method from the marginal variances and sample sizes of each category. In the second stage, trends were combined using random-effects meta-regression. Linear and quadratic models were compared by AIC; non-linearity was assessed via the significance of the quadratic term. Restricted cubic spline models with three knots were not fitted as originally planned, because the small number of contributing cohorts per indicator (two to three) would have resulted in over-parameterization; the more parsimonious quadratic specification was therefore adopted.

## 3. Results

**Figure 1:**
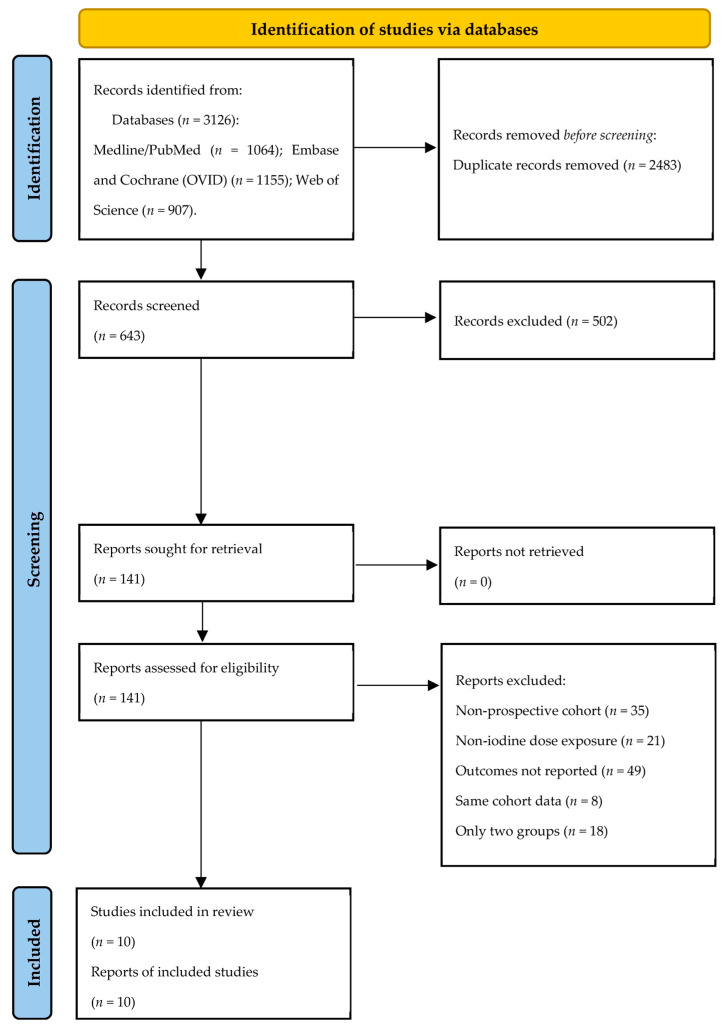
PRISMA diagram of search results and selections.

Of 3126 records initially identified, 2483 were removed as database-level duplicates and 502 were excluded at title-and-abstract screening, leaving 141 full-text reports, all of which were successfully retrieved and assessed for eligibility. Of these, 131 were excluded for the following reasons: 35 were not prospective cohort studies (including cross-sectional analyses, case–control designs, and randomized supplementation trials); 21 did not assess maternal iodine exposure using urinary iodine biomarkers or validated dietary intake instruments (e.g., studies measuring only maternal thyroid hormone levels or household-level iodized-salt access); 49 did not report neurodevelopmental outcomes in the offspring, reported outcomes only at the neonatal period without postnatal follow-up, or reported outcomes that could not be extracted in a form compatible with dose–response meta-analysis (e.g., continuous exposure modeled without categorical effect estimates); 8 were overlapping publications reporting the same outcome data from cohorts already represented by another included publication, and were excluded to prevent double-counting; and 18 reported effect estimates across only two exposure categories, precluding inclusion in the dose–response framework, which required at least three categories. The study selection process is detailed in the PRISMA flow diagram (Figure 1).

### 3.1. Characteristics of the Included Studies

This systematic review and dose–response meta-analysis included a total of 10 original studies encompassing eight prospective cohorts (of which three publications reported different outcome measures from the same cohort), yielding 45 data points after exclusion of the reference categories. Among these, 32 data points (71.1%) reported continuous outcomes (e.g., cognitive scores, language development scores, academic performance), while 13 data points (28.9%) reported binary outcomes (e.g., developmental delay, lowest quartile of IQ). Three publications from the Norwegian Mother and Child Cohort Study [18,19,20] were consolidated under a single cohort label. Because each MoBa publication reported a distinct neurodevelopmental domain, no double-counting occurred within any single pooled estimate. The included studies assessed iodine exposure through dietary intake measurements and urinary iodine concentration (UIC) measurements (Table 1).

Consolidation of the three MoBa publications into a single independent cohort and pre-specification of one primary outcome per cohort–domain combination reduced the number of analytic units to 24 across six independent cohorts for the primary continuous-outcome analysis. For subgroup analysis by exposure indicator, cohorts contributing multiple exposure categories retained all categorical estimates within a single indicator stratum, yielding 15, 4, and 5 estimates for dietary intake, UIC, and UI/Cr, respectively. For binary outcomes, all 13 adjusted odds ratios from four cohorts were retained because no within-cohort duplication of outcome constructs occurred. Sensitivity analysis retains all 32 continuous estimates with cohort-level cluster-robust variance estimation.

### 3.2. Risk of Bias in Studies

The methodological quality of the 10 included cohort studies was evaluated using the Newcastle–Ottawa Scale (NOS; Table 2). Total scores ranged from 6 to 9 (median 8), with nine studies scoring 7 or above and one [18] scoring 6. While these scores formally classify most included studies as low-to-moderate risk of bias under NOS criteria, this instrument-based rating should not be interpreted as indicating uniformly high-quality evidence. Common limitations across the included cohorts—single-time-point iodine measurement, heterogeneous neurodevelopmental instruments across age ranges, and potential residual confounding by maternal intelligence and home environment—constrain the strength of causal inference.

### 3.3. Overall Meta-Analysis by Effect Type

#### 3.3.1. Continuous Outcomes (Domain-Stratified)

**Figure 2:**
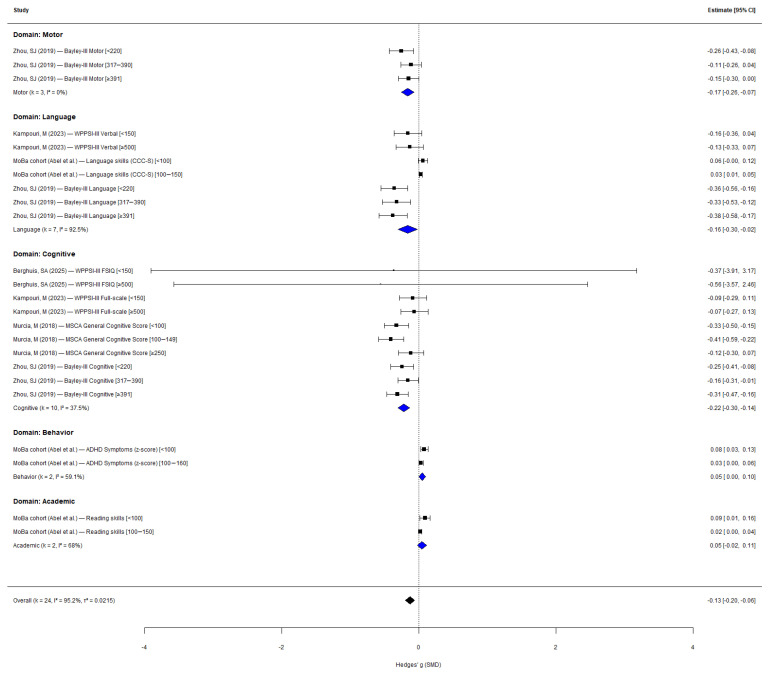
Forest plot of overall meta-analysis for continuous outcomes [18,19,20,21,22,24,26].

After converting all continuous effect estimates to Hedges’ g and selecting one primary outcome per cohort–domain combination, 24 data points from six cohorts across five neurodevelopmental domains were included. The overall pooled effect was statistically significant (Hedges’ g = −0.13, 95% CI: −0.20 to −0.06, *p* < 0.001; Figure 2), indicating that suboptimal maternal iodine status was associated with poorer neurodevelopmental performance. Substantial heterogeneity was observed (*I*^2^ = 95.2%; τ^2^ = 0.022; Q = 143.83, *df* = 23, *p* < 0.001). Although standardization to Hedges’ g permits statistical comparability across instruments, it does not eliminate residual conceptual heterogeneity arising from differences in the cognitive constructs each instrument targets; the pooled estimate should therefore be interpreted as an average standardized effect across a range of neurodevelopmental constructs, rather than as a single unified outcome.

Domain-stratified analysis (Supplementary Figure S1) revealed significant between-domain heterogeneity (QM = 12.92, *df* = 4, *p* = 0.012), explaining 38.6% of the total heterogeneity. Cognitive outcomes showed the largest effect (k = 10; g = −0.22, 95% CI: −0.30 to −0.14, *p* < 0.001; *I*^2^ = 37.5%), followed by motor (k = 3; g = −0.17, 95% CI: −0.26 to −0.07, *p* < 0.001; *I*^2^ = 0%) and language outcomes (k = 7; g = −0.16, 95% CI: −0.30 to −0.02, *p* = 0.026; *I*^2^ = 92.5%). Behavioral outcomes showed a small positive effect reflecting higher ADHD symptom scores with lower iodine (k = 2; g = 0.05, *p* = 0.042), while academic outcomes were non-significant (k = 2; g = 0.05, *p* = 0.178). Within-domain heterogeneity was substantially reduced compared with the overall analysis, with *I*^2^ = 0% for motor and 37.5% for cognitive outcomes.

#### 3.3.2. Binary Outcomes

For binary outcomes, the meta-analysis of 13 data points demonstrated a significant positive association between suboptimal maternal iodine status and increased risk of adverse neurodevelopmental outcomes (pooled OR = 1.19, 95% CI: 1.03 to 1.39, *p* = 0.026; Supplementary Figure S2). Children born to mothers with inadequate iodine status had approximately 19% higher odds of experiencing developmental delays or scoring in the lowest quartile of neurodevelopmental assessments. Heterogeneity was also substantial in this analysis (*I*^2^ = 78.5%, 95% CI: 63.7% to 87.2%; τ^2^ = 0.03; Q = 55.75, *df* = 12, *p* < 0.001).

### 3.4. Subgroup Analysis by Iodine Exposure Indicator

**Figure 3:**
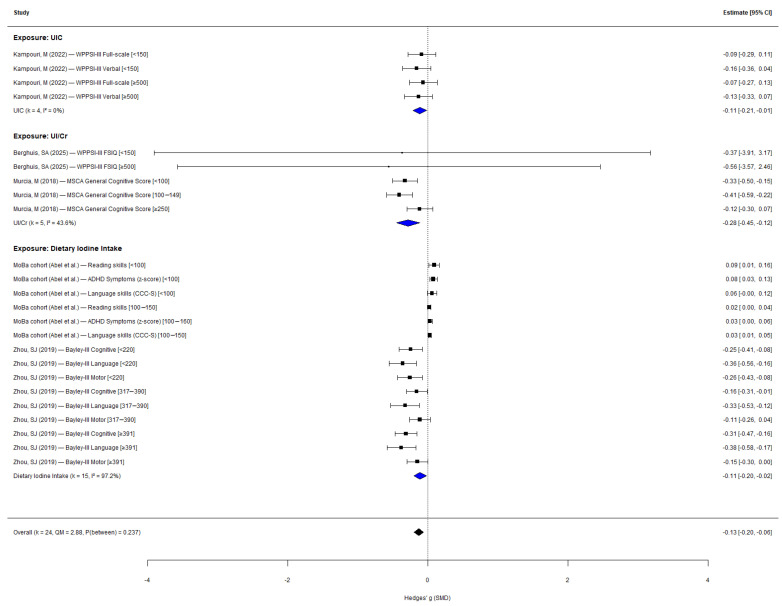
Subgroup analysis by iodine exposure indicator type [18,19,20,21,22,24,26].

Subgroup analysis of continuous outcomes by iodine exposure indicator did not reveal statistically significant between-subgroup differences (QM = 2.88, *p* = 0.237; Figure 3). For studies using dietary iodine intake (k = 15), a small negative effect was observed (g = −0.11, 95% CI: −0.20 to −0.02; *I*^2^ = 97.2%). Studies using UIC (k = 4) showed a similar negative effect (g = −0.11, 95% CI: −0.21 to −0.01; *I*^2^ = 0%). The UI/Cr subgroup (k = 5) showed the largest negative effect (g = −0.28, 95% CI: −0.45 to −0.12; *I*^2^ = 43.6%). Although the between-subgroup test did not reach significance, the UI/Cr subgroup showed notably larger effect sizes, which may reflect the greater precision of creatinine-adjusted urinary iodine as a biomarker of individual-level exposure.

### 3.5. Dose–Response Meta-Analysis

**Figure 4:**
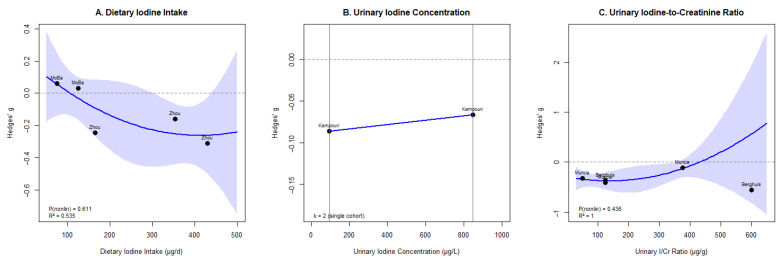
Dose–response relationship between maternal iodine and child neurodevelopment.

Dose–response analyses were conducted separately for each iodine exposure indicator using Hedges’ g, with one primary outcome per cohort. For dietary iodine intake (Figure 4A), data from two cohorts [18,19,20,21] were analyzed. The quadratic model explained 53.5% of variance (R^2^ = 0.535), though non-linearity did not reach significance (*p* = 0.612). The fitted curve displayed a pattern consistent with declining performance at both low (<100 µg/d) and high (>400 µg/d) intakes, approaching its maximum within a mid-range of approximately 150–300 µg/d. This exploratory pattern should not be interpreted as defining a clinically optimal intake range, given the limited number of contributing cohorts and the non-significant quadratic non-linearity term.

For UIC (Figure 4B), data from only one cohort (Kampouri et al.) precluded formal dose–response modeling; the two available data points indicated modest negative associations at both extremes relative to the 150–500 μg/L reference range. For UI/Cr (Figure 4C), data from two cohorts (Berghuis and Murcia) were analyzed; the quadratic model explained all between-study variance (R^2^ = 1.00), but non-linearity was not significant (*p* = 0.436). Across indicators, dose–response patterns were directionally consistent with an inverted U-shaped relationship, though cohort consolidation and standardization to SMD attenuated statistical power for non-linearity testing.

### 3.6. Sensitivity Analysis

Leave-one-out sensitivity analysis (Supplementary Figure S3) demonstrated that the overall pooled effect remained statistically significant regardless of which data point was omitted, with Hedges’ g ranging from −0.14 to −0.12. For binary outcomes (Supplementary Figure S4), the pooled OR ranged from 1.13 to 1.17, maintaining significance throughout. Robust variance estimation using all available continuous outcomes with cluster-robust standard errors yielded a consistent direction of effect (Hedges’ g = −0.13, robust SE = 0.036), though with only six independent cohort clusters, the Satterthwaite degrees of freedom were low (*df* ≈ 3), limiting the power of this sensitivity analysis (*p* = 0.128).

### 3.7. Publication Bias Assessment

**Figure 5:**
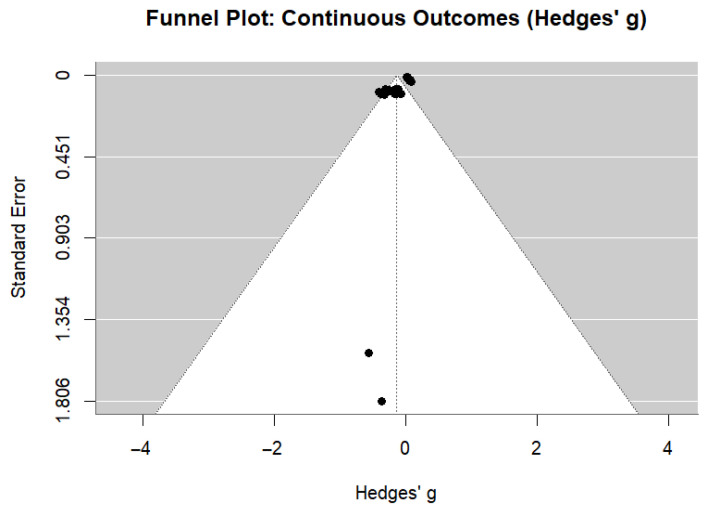
Funnel plot for publication bias assessment (vertical dotted line: pooled effect estimate; filled circles: individual studies; diagonal lines: pseudo-95% confidence interval boundaries).

Visual inspection of the funnel plot for continuous outcomes (Figure 5) revealed asymmetry, confirmed by Egger’s regression test (t = −5.09, *p* < 0.001). The trim-and-fill method imputed six potentially missing studies (Supplementary Figure S5). After adjustment, the pooled effect was attenuated (adjusted g = −0.07, 95% CI: −0.15 to 0.00, *p* = 0.051), suggesting that publication bias may have contributed to effect inflation, though the direction of association persisted.

For binary outcomes (Supplementary Figure S6), the funnel plot showed less pronounced asymmetry. Egger’s test did not reach significance (t = 1.78, *p* = 0.102). The trim-and-fill analysis imputed two studies, yielding an adjusted OR of 1.13 (95% CI: 1.03 to 1.24).

## 4. Discussions

This systematic review and dose–response meta-analysis of 10 prospective cohort studies (eight independent cohorts) provides evidence that maternal iodine status during pregnancy is significantly associated with child neurodevelopmental outcomes. Using standardized mean differences (Hedges’ g) to account for heterogeneous assessment instruments, the pooled analysis demonstrated that suboptimal iodine status was associated with a reduction of 0.13 standard deviations in neurodevelopmental performance (95% CI: −0.20 to −0.06). Domain-stratified analysis revealed that cognitive outcomes were most consistently affected (g = −0.22, *I*^2^ = 37.5%), followed by motor (g = −0.17, *I*^2^ = 0%) and language outcomes (g = −0.16, *I*^2^ = 92.5%), with between-domain heterogeneity explaining 38.6% of the total variance (*p* = 0.012). Within-domain stratification substantially reduced heterogeneity for cognitive and motor outcomes, supporting the use of domain-specific pooling when synthesizing neurodevelopmental data. Nevertheless, SMD standardization addresses scale incompatibility but not conceptual differences between instruments—for example, between Bayley-III cognitive composites at 18 months and WISC-IV full-scale IQ at 10 years—and the domain-stratified estimates should therefore be interpreted as summaries across related but non-identical constructs. Binary outcome analysis confirmed a 15% increase in odds of developmental delay (OR = 1.19, 95% CI: 1.03 to 1.39, *p* = 0.026).

The observed association between maternal iodine status and child neurodevelopment is biologically plausible. Maternal thyroxine (T4) is the primary source of thyroid hormones for the developing fetal brain during the first half of gestation, before fetal thyroid function matures at approximately 18–20 weeks [4,27]. In the fetal cerebral cortex, T4 is locally converted to T3 by type II 5′-iodothyronine deiodinase, and T3 binds nuclear receptors to regulate gene expression essential for neuronal migration, differentiation, synaptogenesis, and myelination [28,29]. Under insufficient maternal iodine intake, the thyroid preferentially synthesizes T3 over T4 to conserve iodine—an adaptation that protects maternal euthyroidism but compromises placental T4 supply to the fetus [5,30]. At the opposite extreme, excessive iodine exposure can transiently suppress thyroid hormone synthesis via the Wolff–Chaikoff effect [12], and both animal and human studies have linked iodine excess to offspring neurodevelopmental impairment and maternal thyroid dysfunction [13,31,32]. Biological plausibility, however, does not by itself constitute evidence of a specific quantitative association in humans; the mechanistic framework outlined above provides a rationale for why maternal iodine status might influence child neurodevelopment, but the magnitude and shape of this association in observational data remain subject to measurement, heterogeneity, and publication-bias limitations.

Our findings are broadly consistent with the individual participant data meta-analysis by Levie et al., which reported that a lower urinary iodine-to-creatinine ratio during pregnancy was associated with reduced verbal IQ in offspring, particularly when measured during the first trimester [15]. However, in our study, we extend that work in several important ways. While Levie et al. analyzed data from only three European cohorts using a two-stage approach with cohort-level iodine-to-creatinine ratios, our analysis incorporated 10 studies from diverse geographic settings (Norway, Australia, Bangladesh, Japan, Canada, the United Kingdom, China, and Spain), enhancing the generalizability of findings across populations with varying baseline iodine status and dietary patterns. Furthermore, our use of a dose–response meta-analytic framework with quadratic dose–response modeling allowed us to characterize the shape of the association across the entire iodine exposure spectrum, suggesting patterns that categorical comparisons in prior reviews could not directly characterize, although our ability to formally demonstrate non-linearity was limited by the small number of contributing cohorts.

In a systematic review of iodine supplementation effects in mildly-to-moderately deficient pregnant women, Taylor et al. concluded that meta-analyses of two RCTs showed no significant effect on child cognitive, language, or motor scores [33]. This apparent discrepancy with our findings likely reflects fundamental differences between supplementation trials and observational dose–response analyses. RCTs of iodine supplementation during pregnancy have generally enrolled women with mild deficiency and often initiated supplementation after the first trimester, potentially missing the most vulnerable developmental window [34,35]. In contrast, observational cohort studies capture the full range of habitual iodine exposure, including severe deficiency, and assess exposure from conception onward, thereby capturing the effects of early gestational iodine status on fetal brain development. Our observational findings and the null trial findings can be viewed as complementary perspectives rather than as conflicting evidence, although the divergence between the two lines of evidence also leaves the causal contribution of maternal iodine status to child neurodevelopment unresolved.

The dose–response analysis, conducted using standardized effect sizes after consolidating overlapping MoBa publications, represents a methodological refinement over the original analysis. By modeling each indicator separately and expressing outcomes as Hedges’ g, we preserved biological interpretability while enabling cross-instrument comparability. The quadratic model for dietary iodine intake explained 53.5% of between-study variance, with the fitted curve approaching its maximum within a descriptive mid-range of approximately 150–300 µg/d. This range overlaps with the WHO recommendation of 250 µg/d [8] and the EAR of 160 µg/d used in the MoBa studies [18,19], but given the non-significant quadratic term and the small number of contributing cohorts, our finding should be regarded as descriptively consistent with existing guidance rather than as independent evidence supporting any specific intake threshold. For UIC, formal dose–response modeling was precluded by the availability of only one contributing cohort after consolidation [22]. Despite these power limitations, the directional patterns across indicators consistently supported an inverted U-shaped relationship, with neurodevelopmental outcomes optimized within a mid-range of exposure and decrements at both extremes. The decision to model each indicator separately remains methodologically important, because directly combining μg/d, μg/L, and μg/g conflates exposures reflecting different aspects of iodine metabolism.

The interpretation of our dose–response findings must also account for the inherent measurement properties of the three iodine indicators. Spot urinary iodine concentration exhibits substantial within-individual variability, with day-to-day coefficients of variation commonly exceeding 30%, owing to its dependence on recent dietary intake and urinary dilution; a single spot measurement therefore reflects transient rather than habitual exposure and introduces non-differential misclassification that attenuates true dose–response relationships toward the null. The urinary iodine-to-creatinine ratio partially mitigates dilution-related variability but remains influenced by muscle mass and short-term diet, while food-frequency-based dietary intake estimates are prone to recall bias and incomplete capture of iodized-salt intake. Collectively, these measurement limitations imply that the apparent optimal ranges identified in our dose–response curves likely represent broader intervals than the point estimates suggest, and that the true shape of the association may be more pronounced than current data can resolve.

The high heterogeneity observed in several analyses and the evidence of publication bias warrant explicit interpretive caution. Although *I*^2^ reached 95.2% in the overall continuous pooled analysis and remained above 90% in the language and dietary-intake subgroups, domain-stratified pooling substantially reduced within-domain heterogeneity for cognitive (*I*^2^ = 37.5%) and motor outcomes (*I*^2^ = 0%), indicating that scale and domain differences—rather than true effect variability—drove much of the between-study inconsistency. Residual heterogeneity in language and dietary-intake analyses likely reflects differences in assessment age, instrument sensitivity, and baseline iodine background across populations, and the pooled estimates in these strata should therefore be interpreted as averages across heterogeneous contexts rather than as universal effect sizes. Egger’s regression indicated significant small-study asymmetry (t = −5.09, *p* < 0.001), and trim-and-fill attenuated the pooled Hedges’ g from −0.13 to −0.07 (95% CI: −0.15 to 0.00), suggesting that the true effect may be closer to the null than the unadjusted estimate implies; this possibility further supports treating the present findings as exploratory.

Our findings carry several implications for clinical guidelines and public health policy. First, the confirmation of a significant association between suboptimal maternal iodine status and adverse neurodevelopmental outcomes reinforces the importance of ensuring adequate iodine nutrition for all pregnant women, particularly in regions where universal salt iodization has not been fully implemented or where its coverage is declining. Second, the descriptive mid-range identified in our exploratory dose–response curves (approximately 150–300 µg/d) is broadly consistent with existing WHO [8] and ATA/ETA [10] recommendations, but does not, in itself, constitute independent evidence supporting any specific intake threshold; existing guidelines should continue to guide clinical practice. Third, the potential for adverse effects at very high iodine levels is consistent with existing cautions against uncritical iodine supplementation in populations with habitually high intake, particularly in populations with habitually high iodine consumption [36], although our data do not permit quantification of the threshold above which adverse effects emerge without concurrent monitoring of excessive intake.

Several directions for future research emerge from our findings. Large-scale prospective cohort studies with serial iodine measurements across pregnancy trimesters are needed to identify trimester-specific vulnerability windows and to strengthen causal inference [18]. More research should be conducted to draw more attention towards regions with iodine excess, such as parts of East Asia where dietary iodine intake is high due to seaweed consumption [37]. Well-powered RCTs of iodine supplementation initiated before or during the first trimester, with long-term neurodevelopmental follow-up, remain a critical evidence gap [33]. Additionally, future studies should standardize iodine exposure assessment using urinary biomarkers corrected for dilution (e.g., iodine-to-creatinine ratio or estimated 24 h urinary iodine excretion), rather than relying solely on spot UIC or dietary questionnaires [38]. Finally, individual participant data meta-analyses incorporating a broader range of cohorts and utilizing harmonized neurodevelopmental outcome measures would provide more precise dose–response estimates and enable the investigation of potential effect modifiers such as maternal thyroid autoimmunity, selenium status, and genetic polymorphisms in thyroid hormone metabolism.

Several limitations should be acknowledged. First, the included studies are observational, and despite adjustment for multiple confounders, residual confounding by unmeasured factors such as maternal intelligence, home learning environment, and co-occurring micronutrient deficiencies cannot be excluded. Second, the reliance on single-time-point iodine measurements in most studies may not fully capture the dynamic changes in iodine status across pregnancy, potentially introducing exposure misclassification. Third, the heterogeneity in neurodevelopmental outcome measures and assessment ages across studies limits the precision of pooled estimates. Fourth, although the use of standardized mean differences (Hedges’ g) and domain-stratified pooling mitigated the impact of scale heterogeneity, residual conceptual heterogeneity across assessment instruments within the same domain (e.g., Bayley-III vs. WPPSI-III for cognitive outcomes) cannot be fully resolved by statistical standardization alone; we therefore provide domain-stratified results to aid interpretation. Fifth, publication bias was detected for continuous outcomes (Egger test *p* < 0.001), and although the trim-and-fill adjusted estimate remained significant (adjusted g = −0.07, 95% CI: −0.15 to 0.00), the attenuation suggests that the true effect may be smaller than the unadjusted pooled estimate. Sixth, the dose–response curve at very high iodine levels (above 500 μg/L) should be interpreted cautiously due to sparse data in this range, and the combining of different iodine indicators (μg/L for UIC and μg/d for dietary intake) into a single dose–response model represents a simplification that may introduce measurement heterogeneity. Seventh, the relatively small number of included studies (k = 10) may limit the statistical power for subgroup and moderator analyses.

## 5. Conclusions

This systematic review and dose–response meta-analysis of 10 prospective cohort publications, corresponding to eight independent cohorts, demonstrates that maternal iodine status during pregnancy is significantly associated with child neurodevelopmental outcomes. After standardizing continuous effect estimates to Hedges’ g and consolidating overlapping publications from the same cohort, suboptimal maternal iodine status was associated with a modest but significant decrement in neurodevelopmental performance (pooled g = −0.13; 95% CI: −0.20 to −0.06), with cognitive outcomes most consistently affected. Exploratory dose–response analyses suggested that the fitted curves approached their maximum within a mid-range of dietary iodine intake (approximately 150–300 µg/d); the quadratic non-linearity terms did not reach statistical significance after cohort consolidation, and the directional patterns across indicators, although consistent with an inverted U-shaped relationship, should be regarded as hypothesis-generating. These exploratory findings do not support the formulation of specific intake recommendations and should not be interpreted as clinical guidance. Because dietary intake, UIC and UI/Cr reflect distinct aspects of iodine metabolism, they should not be interpreted interchangeably. These findings support the maintenance of adequate but not excessive iodine nutrition during pregnancy. Future studies should employ serial trimester-specific measurements, standardized urinary biomarkers, and harmonized neurodevelopmental instruments to refine the dose–response relationship.

## Figures and Tables

**Table 1:** Characteristics of the included prospective cohort studies.

Study ID (Author, Year)	Country	Sample Size (N)	Population Iodine Background (Median UIC < 150/150–249/≥300 μg/L)	Iodine Exposure Indicator	Reference Group Definition	Follow-Up
Abel, M.H. et al. (2019) [19]	Norway	39,471	Mild–Moderate Deficient	Iodine Intake from Food (FFQ)	Iodine intake from food ≥ 150 μg/d (for trend)	8 years
Zhou, S.J. et al. (2019) [21]	Australia	699	Sufficient	Iodine Intake from FFQ and UIC	Iodine Intake Q2 (220–316 μg/d); UIC ≥ 150 μg/L	18 months
Kampouri, M. et al. (2023) [22]	Bangladesh	1530	Above Adequate/Excessive	Urinary Iodine Concentration (UIC)	150 ≤ UIC < 500 μg/L (pregnancy); 100 ≤ UIC < 300 μg/L (childhood)	5 and 10 years
Abel, M.H. et al. (2017) [18]	Norway	48,297	Mild–Moderate Deficient	Iodine Intake from Food (FFQ)	Iodine intake 160 μg/d (EAR)	3 years
Abel, M.H. et al. (2017) [20]	Norway	77,164	Mild–Moderate Deficient	Iodine Intake from Food and Supplements	Iodine intake 160 μg/d (EAR)	8 years
Hisada, A. et al. (2022) [23]	Japan	75,249 (1y); 66,604 (3y)	Sufficient (but with wide range)	Iodine Intake from FFQ (μg/d)	Intake Q4 (176–276 μg/d)	1 and 3 years
Berghuis, S.A. et al. (2025) [24]	Canada	1501 (thyroid); 760 (neurodev)	Sufficient	Urinary Iodine-to-Creatinine Ratio (UI/Creat)	UI/Creat 150–500 μg/g	3–4 years
Bath, S.C. et al. (2013) [7]	UK	958	Mild–Moderate Deficient	Urinary Iodine-to-Creatinine Ratio (UI/Creat)	UI/Creat ≥ 150 μg/g	8–9 years
Wu, W. et al. (2023) [25]	China	469	Sufficient	Urinary Iodine Concentration (UIC)	UIC 150–249 μg/L	18–24 months
Murcia, M. et al. (2018) [26]	Spain	1803	Mild–Moderate Deficient	Iodine Intake, Supplements, UIC, UIC/Cr	UIC/Cr 150–249 μg/g	4–5 years

Full baseline characteristics, including whole-population median UIC, universal salt-iodization status, and detailed exposure category definitions, are provided in Supplementary Table S1.

**Table 2:** Detailed risk-of-bias assessment of included cohort studies using the Newcastle–Ottawa Scale (NOS).

Study (Author, Year)	S1: Representativeness	S2: Selection of Non-Exposed	S3: Ascertainment of Exposure	S4: Outcome Not Present at Start	C1: Controls for Most Important Factor	C2: Controls for Additional Factors	O1: Assessment of Outcome	O2: Follow-Up Long Enough	O3: Adequacy of Follow-Up	Total Score (Max 9)
Abel et al. 2019 [19]	0	1	1	1	1	1	1	1	0	7
Zhou et al. 2019 [21]	1	1	1	1	1	1	1	1	1	9
Kampouri et al. 2023 [22]	1	1	1	1	1	1	1	1	1	9
Abel et al. 2017 [18]	0	1	1	1	1	1	0	1	0	6
Abel et al. 2017 [20]	0	1	1	1	1	1	1	1	0	7
Hisada et al. 2022 [23]	1	1	0	1	1	1	0	1	1	7
Berghuis et al. 2025 [24]	1	1	1	1	1	1	1	1	1	9
Bath et al. 2013 [7]	0	1	1	1	1	1	1	1	0	7
Wu et al. 2023 [25]	0	1	1	1	1	0	1	1	1	7
Murcia et al. 2018 [26]	0	1	1	1	1	1	1	1	1	8

## Data Availability

The data that support the findings of this study are available from the corresponding author upon reasonable request.

## Supplementary Materials

The following supporting information can be downloaded at https://www.mdpi.com/article/10.3390/nu18091474/s1, File S1: Search strategy; File S2: Statistical formulas for effect-size standardization and two-stage dose–response meta-analysis; Table S1: Full baseline characteristics; Figure S1: Forest Plot for Binary Outcomes; Figure S2: Domain-stratified forest plot of continuous outcomes; Figure S3: Leave-One-Out Sensitivity Analysis; Figure S4: Leave-One-Out Sensitivity Analysis for Binary Outcomes; Figure S5: Funnel Plot with Trim-and-Fill Adjustment; Figure S6: Funnel Plot for Binary Outcomes.
