# Supplementary material for: Maternal Iodine Status During Pregnancy and Child Neurodevelopment: A Systematic Review and Dose–Response Meta-Analysis of Prospective Cohort Studies
Source: Nutrients. 2026 May 5;18(9):1474. doi: 10.3390/nu18091474 (PMC13165351; doi:10.3390/nu18091474)
Supplement: Supplementary file 1 [file nutrients-18-01474-s001.zip › nutrients-4252115-supplementary.pdf]

Supplementary File S1: Search strategy

1. PubMed Search Strategy

No.	Search Strategy
#1	"Iodine"[MeSH Terms]
#2	"Iodides"[MeSH Terms]
#3	"iodine"[Title/Abstract]
#4	"iodide"[Title/Abstract] OR "iodides"[Title/Abstract]
#5	"urinary iodine"[Title/Abstract]
#6	"iodine concentration"[Title/Abstract]
#7	"iodine intake"[Title/Abstract]
#8	"iodine status"[Title/Abstract]
#9	"iodine nutrition"[Title/Abstract]
#10	"iodine deficiency"[Title/Abstract] OR "iodine excess"[Title/Abstract]
#11	"UIC"[Title/Abstract]
#12	"iodine-to-creatinine"[Title/Abstract] OR "iodine creatinine ratio"[Title/Abstract]
#13	"iodized salt"[Title/Abstract] OR "iodised salt"[Title/Abstract]
#14	#1 OR #2 OR #3 OR #4 OR #5 OR #6 OR #7 OR #8 OR #9 OR #10 OR #11 OR #12 OR #13
#15	"Pregnancy"[MeSH Terms]
#16	"Pregnant Women"[MeSH Terms]
#17	"Prenatal Exposure Delayed Effects"[MeSH Terms]
#18	"Maternal Exposure"[MeSH Terms]
#19	"pregnan*"[Title/Abstract]
#20	"maternal"[Title/Abstract]
#21	"gestation*"[Title/Abstract]
#22	"prenatal"[Title/Abstract] OR "antenatal"[Title/Abstract]
#23	"periconceptional"[Title/Abstract] OR "periconception*"[Title/Abstract]
#24	"trimester"[Title/Abstract]
#25	#15 OR #16 OR #17 OR #18 OR #19 OR #20 OR #21 OR #22 OR #23 OR #24
#26	"Child Development"[MeSH Terms]
#27	"Neurodevelopmental Disorders"[MeSH Terms]
#28	"Cognition"[MeSH Terms]
#29	"Intelligence"[MeSH Terms]
#30	"Language Development"[MeSH Terms]
#31	"Motor Skills"[MeSH Terms]
#32	"Intellectual Disability"[MeSH Terms]

#33	"Child Behavior"[MeSH Terms]
#34	"neurodevelopment*"[Title/Abstract]
#35	"cognitive"[Title/Abstract] OR "cognition"[Title/Abstract]
#36	"intelligence"[Title/Abstract] OR "IQ"[Title/Abstract]
#37	"language development"[Title/Abstract] OR "language delay"[Title/Abstract]
#38	"motor development"[Title/Abstract] OR "motor skills"[Title/Abstract]
#39	"mental development"[Title/Abstract] OR "psychomotor"[Title/Abstract]
#40	"Bayley"[Title/Abstract] OR "BSID"[Title/Abstract]
#41	"MDI"[Title/Abstract] OR "PDI"[Title/Abstract]
#42	"developmental delay"[Title/Abstract]
#43	"academic performance"[Title/Abstract] OR "school performance"[Title/Abstract]
#44	"attention deficit"[Title/Abstract] OR "ADHD"[Title/Abstract]
#45	"behavio*"[Title/Abstract] AND "child*"[Title/Abstract]
#46	#26 OR #27 OR #28 OR #29 OR #30 OR #31 OR #32 OR #33 OR #34 OR #35 OR #36 OR #37 OR #38 OR #39 OR #40 OR #41 OR #42 OR #43 OR #44 OR #45
#47	#14 AND #25 AND #46

2. Embase and Cochrane Library Search Strategy (OVID)

No.	Search Strategy
1	exp iodine/
2	exp iodide/
3	exp iodine deficiency/
4	(iodine or iodide or iodides).ti,ab,kw.
5	(urinary iodine or iodine concentration).ti,ab,kw.
6	(iodine intake or iodine status or iodine nutrition).ti,ab,kw.
7	(iodine deficiency or iodine excess).ti,ab,kw.
8	UIC.ti,ab,kw.
9	(iodine-to-creatinine or iodine creatinine ratio).ti,ab,kw.
10	(iodized salt or iodised salt).ti,ab,kw.
11	1 or 2 or 3 or 4 or 5 or 6 or 7 or 8 or 9 or 10
12	exp pregnancy/
13	exp pregnant woman/
14	exp prenatal exposure/
15	exp maternal exposure/
16	(pregnan* or maternal or gestation*).ti,ab,kw.
17	(prenatal or antenatal or periconception*).ti,ab,kw.
18	trimester.ti,ab,kw.

19	12 or 13 or 14 or 15 or 16 or 17 or 18
20	exp child development/
21	exp neurodevelopment/
22	exp cognition/
23	exp intelligence/
24	exp language development/
25	exp motor development/
26	exp intellectual impairment/
27	exp child behavior/
28	(neurodevelopment* or cognitive or cognition or intelligence or IQ).ti,ab,kw.
29	(language development or language delay or motor development or motor skills).ti,ab,kw.
30	(mental development or psychomotor).ti,ab,kw.
31	(Bayley or BSID or MDI or PDI).ti,ab,kw.
32	(developmental delay or academic performance or school performance).ti,ab,kw.
33	(attention deficit or ADHD).ti,ab,kw.
34	(behavio* adj3 child*).ti,ab,kw.
35	20 or 21 or 22 or 23 or 24 or 25 or 26 or 27 or 28 or 29 or 30 or 31 or 32 or 33 or 34
36	11 and 19 and 35
37	exp animals/ not exp humans/
38	36 not 37

3. Web of Science Search Strategy

No.	Search Strategy
#1	TS=("iodine" OR "iodide" OR "iodides" OR "urinary iodine" OR "iodine concentration" OR "iodine intake" OR "iodine status" OR "iodine nutrition" OR "iodine deficiency" OR "iodine excess")
#2	TS=("UIC" OR "iodine-to-creatinine" OR "iodine creatinine ratio" OR "iodized salt" OR "iodised salt")
#3	#1 OR #2
#4	TS=("pregnan*" OR "maternal" OR "gestation*" OR "prenatal" OR "antenatal" OR "periconception*" OR "trimester")
#5	TS=("neurodevelopment*" OR "cognitive" OR "cognition" OR "intelligence" OR "IQ" OR "language development" OR "language delay")
#6	TS=("motor development" OR "motor skills" OR "mental development" OR "psychomotor" OR "Bayley" OR "BSID" OR "MDI" OR "PDI")
#7	TS=("developmental delay" OR "academic performance" OR "school performance" OR "attention deficit" OR "ADHD" OR "child behavio*")
#8	#5 OR #6 OR #7
#9	#3 AND #4 AND #8

Supplementary File S2. Statistical formulas for effect-size standardization and two-stage dose–response meta-analysis

A. Standardized mean difference (Hedges' g)

For each exposure category i in study k, the standardized mean difference was computed as follows. When the included study reported a regression coefficient on a raw-score scale (e.g., Bayley-III points, WPPSI-III full-scale IQ points), the raw coefficient β_{ik} was standardized by the instrument-specific population standard deviation SD_e:

$$g_{ik} = J \times (\beta_{ik} / SD_e)$$

When the coefficient was already reported on a z-score-transformed outcome, β_{ik} was interpreted directly as the standardized effect ($SD_e = 1$). The Hedges small-sample correction factor J was applied to all estimates:

$$J = 1 - 3 / (4 \times df - 1)$$

where df denotes the residual degrees of freedom of the underlying regression model. Sampling variances of g_{ik} were derived analogously from the reported standard errors or confidence intervals and multiplied by J^2.

B. Greenland–Longnecker covariance reconstruction

For studies reporting effect estimates across multiple ordered exposure categories from the same participants, the within-study covariance between categories was reconstructed following Greenland and Longnecker [16]. For two non-reference categories i and j sharing a common reference category r, the covariance is:

$$Cov(g_i, g_j) = s^2 \times (1/n_r) \times J^2$$

where s^2 is the pooled within-category variance approximated from the reported category-specific standard errors and sample sizes, and n_r is the sample size of the reference category. The full K × K covariance matrix (where K is the number of non-reference categories) was constructed accordingly and used as the weight matrix in the first-stage generalized least squares regression.

C. Two-stage dose–response meta-regression

In the first stage, a study-specific dose–response trend was fitted using generalized least squares:

$$g_{ik} = \beta_{1k} \times x_{ik} + \beta_{2k} \times x^2_{ik} + \varepsilon_{ik}$$

where x_{ik} denotes the assigned dose for category i in study k (midpoint of the exposure range), and the error term uses the reconstructed covariance matrix from Section B. In the

second stage, study-specific coefficients β_{1k} and β_{2k} were combined across studies using random-effects meta-regression with REML:

$$\beta\_pooled = \Sigma w_k \times \beta_k / \Sigma w_k, \text{ where } w_k = 1 / (v_k + \tau^2)$$

Non-linearity was assessed by testing whether the pooled quadratic coefficient β_2,pooled differed significantly from zero (two-sided Wald test). Linear and quadratic models were compared by Akaike's Information Criterion, with lower AIC indicating better balance between fit and parsimony.

Supplementary Table S1 Full baseline characteristics

**Study ID (Author, Year)**	**Country**	**Sample Size (N)**	**Age (SD)**	**Population Iodine Background (Median UIC <150 / 150-249 / ≥300 µg/L)**	**Whole Population Median UIC (µg/L)**	**Iodized Salt Policy**	**Iodine Exposure Indicator**	**Reference Group Definition**	**Follow-up**
**Abel, M. H., et al. (2019)[28]**	Norway	39,471	30.6 (4.5)	Mild-Moderate Deficient	Iodine from food: 122 (IQR 89-161); UIC in subsample: 67	Voluntary / Not mandatory (at study time)	Iodine Intake from Food (FFQ)	Iodine intake from food ≥150 µg/d (for trend)	8 years
**Zhou, S. J., et al. (2019)[33]**	Australia	699	30 (5)	Sufficient	Median UIC: 186 (IQR 108-308)	Mandatory Fortification (since 2009)	Iodine Intake from FFQ and UIC	Iodine Intake Q2 (220-316 µg/d); UIC ≥150 µg/L	18 months
**Kampouri, M., et al. (2022)[29]**	Bangladesh	1530	26.5 (6)	Above Adequate / Excessive	Pregnancy: 282; 5y: 406; 10y: 294	Mandatory (since 1989)	Urinary Iodine Concentration (UIC)	150 ≤ UIC <500 µg/L (pregnancy); 100 ≤ UIC <300 µg/L (childhood)	5 and 10 years
**Abel, M. H., et al. (2017)a[27]**	Norway	48,297	30.4 (4.4)	Mild-Moderate Deficient	Iodine from food: 122 (IQR 89-161)	Voluntary / Not mandatory (at study time)	Iodine Intake from Food (FFQ)	Iodine intake 160 µg/d (EAR)	3 years
**Abel, M. H., et al. (2017)b[34]**	Norway	77,164	30.2 (4.5)	Mild-Moderate Deficient	Iodine from food: 121 (IQR 89-162)	Voluntary / Not mandatory (at study time)	Iodine Intake from Food and Supplements	Iodine intake 160 µg/d (EAR)	8 years
**Hisada, A., et al. (2022)[35]**	Japan	75,249 (1y); 66,604 (3y)	29.9	Sufficient (but with wide range)	158 (IQR 50-226)	Not mandatory	Iodine Intake from FFQ (µg/d)	Intake Q4 (176-276 µg/d)	1 and 3 years
**Berghuis, S. A., et al. (2025)[36]**	Canada	1501 (thyroid); 760 (neurodev)	32.3 (5)	Sufficient	310.5 (IQR 216.2-446.6) (UI/Cr)	Not reported (high supplement use)	Urinary Iodine-to-Creatinine Ratio (UI/Creat)	UI/Creat 150-500 µg/g	3-4 years
**Bath, S. C., et al. (2013)[7]**	UK	958	29.8 (4.5)	Mild-Moderate Deficient	91.1 (IQR 53.8-143)	Not mandatory	Urinary Iodine-to-Creatinine Ratio (UI/Creat)	UI/Creat ≥150 µg/g	8-9 years
**Wu, W., et al. (2023)[37]**	China	469	28.6 (3.1)	Sufficient	161 (IQR 110.5-251.4)	Mandatory	Urinary Iodine Concentration (UIC)	UIC 150-249 µg/L	18-24 months
**Murcia, M., et al. (2018)[38]**	Spain	1803	NA	Mild-Moderate Deficient	123 (IQR 73-208)	Not reported	Iodine Intake, Supplements, UIC, UIC/Cr	UIC/Cr 150-249 µg/g	4-5 years

Supplementary Figures S1 Forest Plot for Binary Outcomes

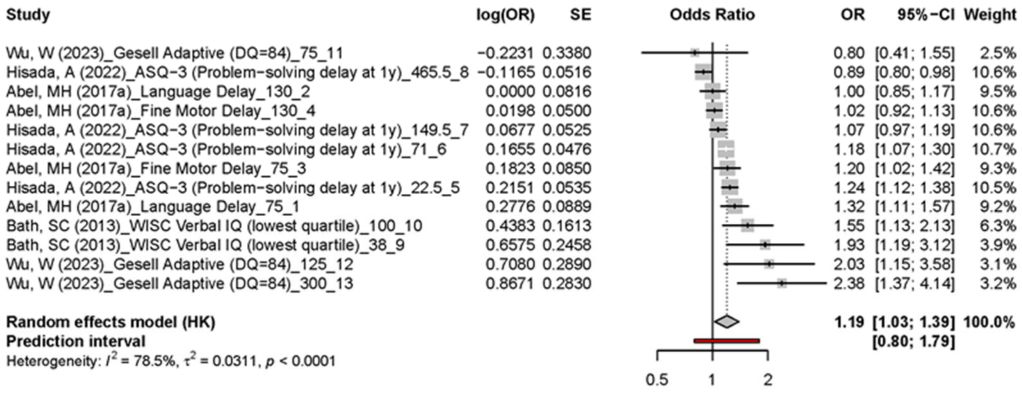

Each row represents one adjusted odds ratio for an adverse neurodevelopmental outcome—developmental delay, lowest-quartile verbal IQ, or ASQ-3 problem-solving delay—in one iodine exposure category versus the cohort-specific reference category. Thirteen effect estimates from four independent cohorts (MoBa, JECS, ALSPAC, Wu-Shanghai) contributed to the pooled random-effects OR of 1.19 (95% CI 1.03–1.39). Suboptimal maternal iodine status encompasses both insufficient (e.g., UIC <150 µg/L, UI/Cr <150 µg/g, dietary intake <160 µg/d) and potentially excessive (e.g., UIC ≥500 µg/L) categories relative to each cohort's reference.

Supplementary Figures S2 Domain-stratified forest plot of continuous outcomes

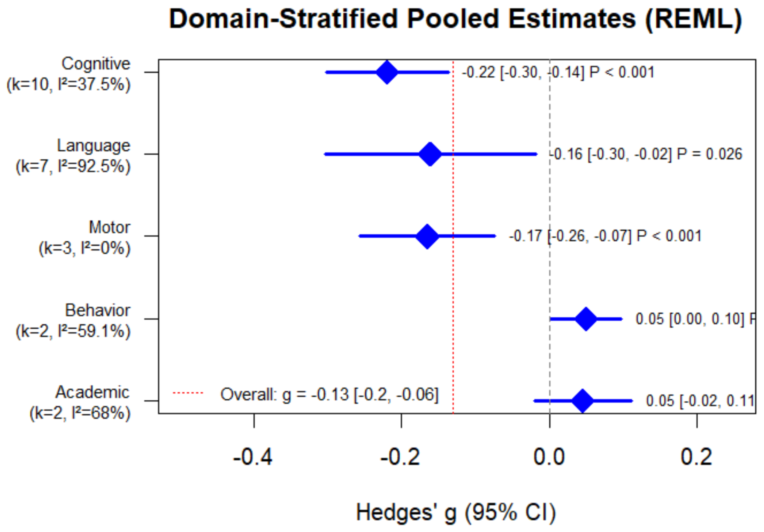

Each row represents the pooled Hedges' g for one neurodevelopmental domain (cognitive, language, motor, behavior, academic), obtained by random-effects meta-analysis (REML) of all primary effect estimates within that domain across cohorts. k denotes the number of contributing effect estimates; one primary outcome per cohort–domain combination was pre-specified to minimize within-cohort replication. Suboptimal maternal iodine status is defined as exposure categories below or above the cohort-specific reference range. Positive g values in the behavior and academic strata arise from scales on which higher scores indicate greater impairment (ADHD symptom z-scores, reading/writing difficulty z-scores) and are therefore interpreted as adverse.

Supplementary Figures S3 Leave-One-Out Sensitivity Analysis

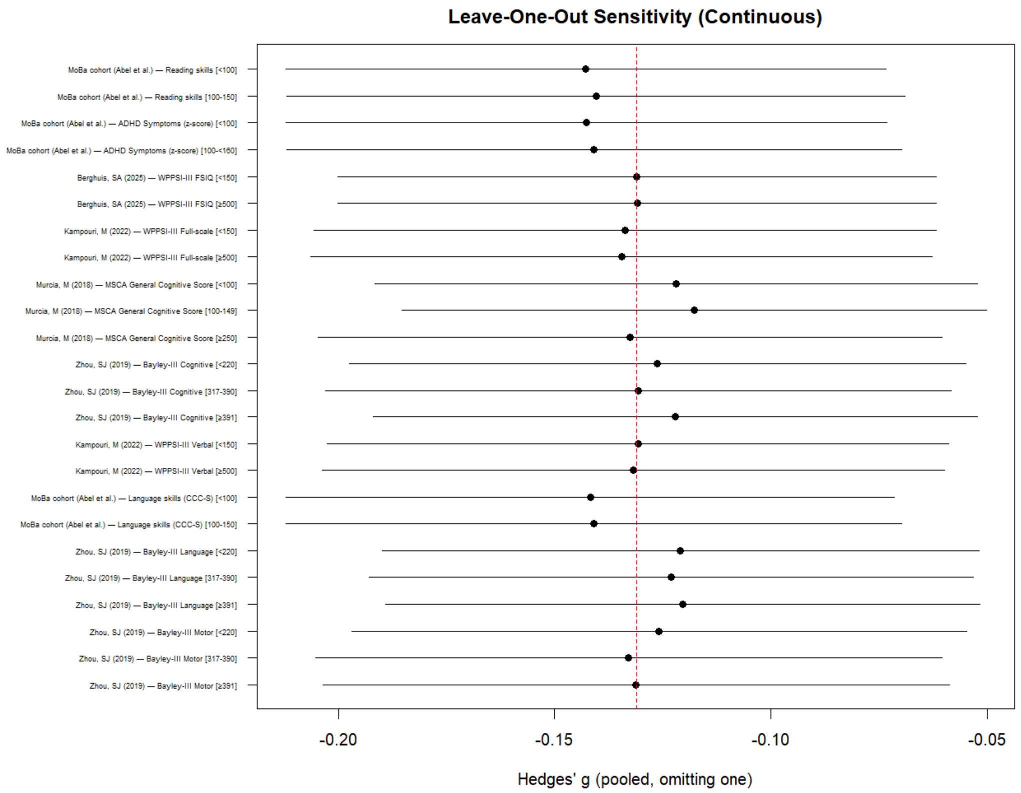

Each point represents the pooled Hedges' g re-estimated after iteratively omitting one effect estimate from the primary domain-stratified analysis. The narrow range of pooled estimates (g = −0.14 to −0.12) indicates that no single data point disproportionately drives the overall finding, supporting the robustness of the pooled effect.

Supplementary Figures S4 Leave-One-Out Sensitivity Analysis for Binary Outcomes

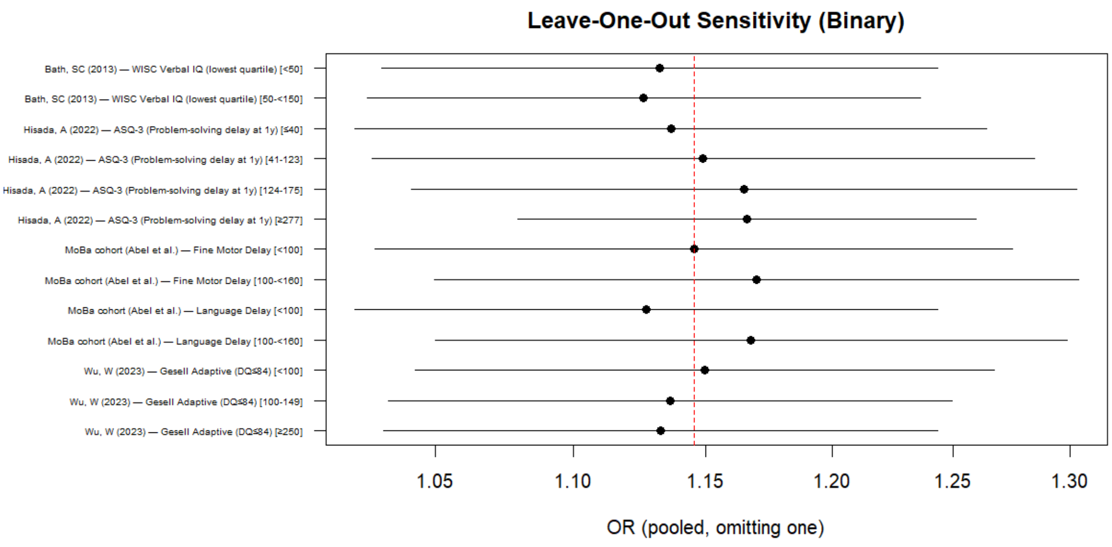

Each point represents the pooled odds ratio re-estimated after iteratively omitting one of the 13 binary effect estimates. The pooled OR ranged from 1.13 to 1.17 across iterations, maintaining statistical significance throughout and confirming that the binary-outcome finding is not driven by any single cohort or effect estimate.

Supplementary Figures S5 Funnel Plot with Trim-and-Fill Adjustment

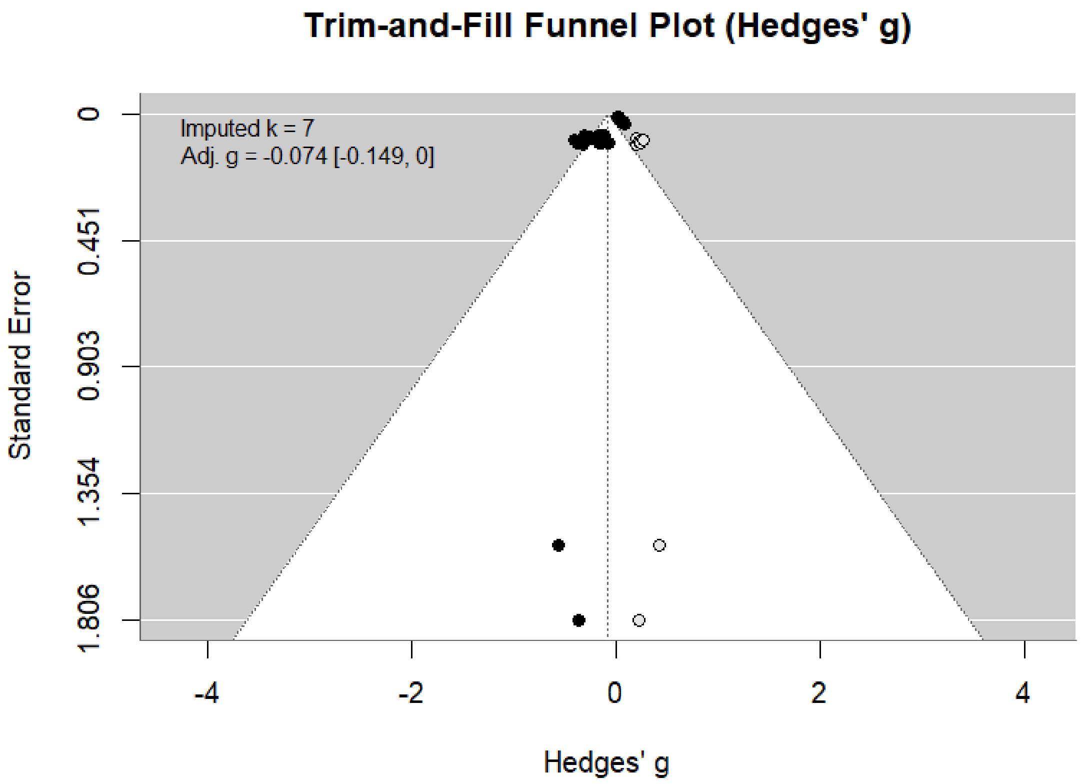

Open circles denote observed effect estimates plotted against their standard errors; solid circles denote hypothetical missing estimates imputed by the Duval–Tweedie trim-and-fill algorithm to correct funnel-plot asymmetry. Egger's regression detected significant asymmetry ($t = -5.09$, $P < 0.001$), and trim-and-fill imputed seven hypothetical studies. The adjusted pooled Hedges' g of −0.07 (95% CI −0.15 to 0.00) was attenuated relative to the unadjusted estimate of −0.13, suggesting that publication bias may have contributed to effect inflation and that the true effect may lie closer to the null.

Supplementary Figures S6 Funnel Plot for Binary Outcomes

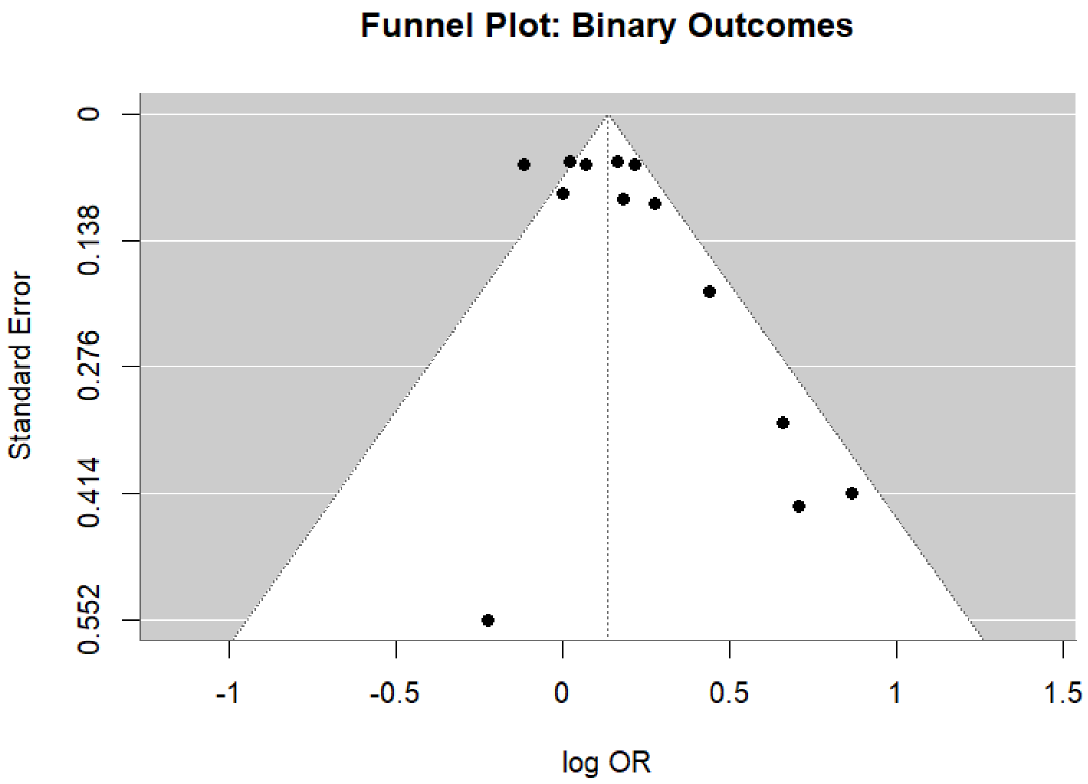

Each point represents the log odds ratio of one binary effect estimate (n = 13) plotted against its standard error. Visual asymmetry was less pronounced than for continuous outcomes; Egger's regression did not reach statistical significance (t = 1.78, P = 0.102). Trim-and-fill imputed two hypothetical studies, yielding an adjusted pooled OR of 1.13 (95% CI 1.03–1.24) and indicating limited evidence of small-study effects in the binary analysis.
